# Research on the Computational Prediction of Essential Genes

**DOI:** 10.3389/fcell.2021.803608

**Published:** 2021-12-06

**Authors:** Yuxin Guo, Ying Ju, Dong Chen, Lihong Wang

**Affiliations:** ^1^ Yangtze Delta Region Institute (Quzhou), University of Electronic Science and Technology of China, Quzhou, China; ^2^ Key Laboratory of Computational Science and Application of Hainan Province, Haikou, China; ^3^ Key Laboratory of Data Science and Intelligence Education, Hainan Normal University, Ministry of Education, Haikou, China; ^4^ School of Mathematics and Statistics, Hainan Normal University, Haikou, China; ^5^ School of Informatics, Xiamen University, Xiamen, China; ^6^ College of Electrical and Information Engineering, Quzhou University, Quzhou, China; ^7^ Beidahuang Industry Group General Hospital, Harbin, China

**Keywords:** communication coding, database, essential genes, machine learning, minimum feature group

## Abstract

Genes, the nucleotide sequences that encode a polypeptide chain or functional RNA, are the basic genetic unit controlling biological traits. They are the guarantee of the basic structures and functions in organisms, and they store information related to biological factors and processes such as blood type, gestation, growth, and apoptosis. The environment and genetics jointly affect important physiological processes such as reproduction, cell division, and protein synthesis. Genes are related to a wide range of phenomena including growth, decline, illness, aging, and death. During the evolution of organisms, there is a class of genes that exist in a conserved form in multiple species. These genes are often located on the dominant strand of DNA and tend to have higher expression levels. The protein encoded by it usually either performs very important functions or is responsible for maintaining and repairing these essential functions. Such genes are called persistent genes. Among them, the irreplaceable part of the body’s life activities is the essential gene. For example, when starch is the only source of energy, the genes related to starch digestion are essential genes. Without them, the organism will die because it cannot obtain enough energy to maintain basic functions. The function of the proteins encoded by these genes is thought to be fundamental to life. Nowadays, DNA can be extracted from blood, saliva, or tissue cells for genetic testing, and detailed genetic information can be obtained using the most advanced scientific instruments and technologies. The information gained from genetic testing is useful to assess the potential risks of disease, and to help determine the prognosis and development of diseases. Such information is also useful for developing personalized medication and providing targeted health guidance to improve the quality of life. Therefore, it is of great theoretical and practical significance to identify important and essential genes. In this paper, the research status of essential genes and the essential genome database of bacteria are reviewed, the computational prediction method of essential genes based on communication coding theory is expounded, and the significance and practical application value of essential genes are discussed.

## Introduction

With the smooth progress of the Human Genome Project, more and more new genes have been discovered, and the study of gene function has become a major issue in the field of life sciences. Genes support the basic structure and performance of life. It stores all the information about the race, blood type, gestation, growth, apoptosis and other processes of life. The environment and genetics jointly determine important physiological processes such as reproduction, cell division, and protein synthesis. Genes are involved in diverse phenomena such as birth, growth, decline, illness, aging, and death (Yang et al., 2015; Hu et al., 2020; Cheng et al., 2021a). Except for some viruses whose genes are composed of ribonucleic acid (RNA) (Sun et al., 2013; Riaz and Li, 2019), in most organisms, genes are composed of deoxyribonucleic acid (DNA) arranged linearly on chromosomes. The existence of a simple life form requires at least 265 to 350 genes, whose functions are affected by internal factors and external environmental factors.

The impact of human genome research on medicine has gradually entered the clinic and changed the traditional medical model. Human genomic research has had a major impact on medicine. Genomic medicine will gradually be applied in clinical practice, and will change the traditional medical model (Liu et al., 2016a; Zhou et al., 2019; Zou, 2019; Chen et al., 2020a; Liu et al., 2020a; Li et al., 2021; Qi et al., 2021). Many believe that it will trigger the next medical revolution (Zeng et al., 2022). Genomic medicine aims to prevent diseases and apply specific gene therapies to address existing genetic defects. In-depth clinical genomic research heralds the arrival of a new era of medicine with a focus on drug optimization, and the prediction and prevention of diseases based on the genome of each patient. Genomic research has identified preferred targets for the development of new drugs (Grazziotin et al., 2015; Yan et al., 2019; Yu et al., 2020a; Zeng et al., 2020a; Liu et al., 2020b; Zhang et al., 2020; Zhuang et al., 2020; Yan et al., 2021a; Yan et al., 2021b; Deng et al., 2021; Shang et al., 2021).

So far, there are dozens of experimental methods to identify essential genes. The methods to realize essential gene identification are mainly divided into two kinds, one is to determine by means of wet experiment; The second is to use computational biology to predict the necessary genes identified by experiments. Wet experiments include: In 1995, Itaya used induced mutations to detect essential genes of *Bacillus subtilis*, Venter used global transposon mutations to identify essential genes of *Mycoplasma genitalium*, targeted gene knockout, transposon mutations, genetic imprinting, and scattered Shotgun method, RNA interference and CRISPR technology (Liu et al., 2016b; Fang et al., 2019a; Ru et al., 2019; Chen et al., 2021). And some researchers have made progress in different fields through the study of essential genes using different wet experiments. Such as: Uddin et al. conducted a comparative genomics analysis and docking studies on *Acinetobacter baumannii*, and predicted and analyzed its non-host essential genes to screen for new drug candidates (Cheng et al., 2018; Uddin et al., 2019; Wang et al., 2020). Wang et al. used the CRISPR (clustered regularly interspaced short palindromic repeats) system to construct a genome-wide single-guide RNA library and screened for genes essential for the survival and proliferation of human cancer cell lines, thus providing a cancer cell identification strategy (Lander et al., 2015). In the field of energy and chemical industries, Voshol et al. created a transposons library of *Synechococcus elongatus* PCC 7942, and then screened it to identify new target genes. The aim was to improve the production capacity of fatty acids and hydrocarbons, and the gene encoding the GTP-binding protein Era was identified as an essential gene in this process (Voshol et al., 2015; Hu et al., 2021a). Another research group suppressed genes in the chloroplasts of *Chlamydomonas* to study its essential signaling pathways, regulatory circuits, and gene functions (Rochaix and Ramundo, 2015; Fang et al., 2019b; Cheng et al., 2020; Wang et al., 2021a). Such analyses can shed light on the growth and photosynthetic processes of photosynthetic organisms, so that strategies can be developed to enhance the photosynthetic ability of engineered bacteria. Therefore, studies on essential genes have theoretical significance and also have practical application value. Therefore, it is of great theoretical significance and practical value to study essential genes.

The disadvantages of wet experiments are that they are expensive, time-consuming, inconsistent in their accuracy, and they can give different experimental results. In addition, wet experimental methods are not applicable to some bacteria. At the same time, as research progresses, it is necessary to obtain essential genes on a genome-wide scale in order to obtain as complete a set of data as possible. This presents a serious challenge to the determination of essential genes by wet assay (Yang et al., 2014; Tavasolian et al., 2020). Therefore, some scientists began to establish predictive models with higher accuracy based on the essential gene information and the biological characteristics of essential genes, combined with computer science and various mathematical algorithms, so as to realize the rapid identification of essential genes. Therefore, greatly reducing unnecessary time and capital consumption.

## Essential Gene Database

Bacteria are abundant both in terms of the number of species and the size of populations. Only a small proportion of bacteria have been fully sequenced. Currently, the following databases provide essential genetic data:

1) NCBI: Holds genomic data for more than 35,000 bacteria. Essential/non-essential genes have been fully determined for only a few bacteria.
2) The Database of Essential Genes (DEG): This database has been developed and maintained by Tianjin University. The latest version (DEG 15.2) contains essential genetic information for 41 kinds of bacteria (48 groups) (Luo et al., 2014), 26 essential gene datasets for eukaryotes and one essential gene dataset for archaea.
3) CEG (Cluster of Essential Genes): Holds information about essential homologous gene clusters for 29 kinds of bacteria (Ye et al., 2013).
4) OGEE (Online Gene Essentiality Database): Holds information about essential genes for 39 kinds of bacteria (Chen et al., 2011).
5) PEC database: Holds information for essential genes in *Escherichia coli*, including relevant structural information and gene function information (Rivas et al., 2015).

## Methods for Identifying Essential Genes

The study of essential genes can also obtain potential drug targets, which can be used to develop antibacterial drugs to resist the invasion of pathogenic microorganisms. Therefore, it is of great practical value to study the theoretical and computational prediction methods of essential genes. Theoretical prediction is a common method of comparative genomics, which is to understand the characteristics of genes to be tested by comparing with the structure of known genes. The disadvantage of this method is that it can not accurately determine all the necessary genes of a certain microorganism, and it is more difficult to analyze eukaryotes. Computational prediction is a potential computational prediction, which uses a variety of biological characteristics of the research object, combined with statistical and classification prediction algorithms, to predict essential genes. With the rapid development of technology, omics data such as protein structure and interaction are widely used in essential gene prediction analysis. Therefore, computational biology methods can be used to provide suitable candidate target genes. The main steps are as follows: First, algorithms and tools are developed to predict essential genes of pathogenic bacteria through computational modeling. This step yields a set of candidate essential genes. Second, an essential gene prediction model is constructed. The model includes information on conserved regions of genes, and this allows for the identification of essential genes showing homology among multiple species. Next, the sequences of the obtained essential genes are compared with the genome sequences of humans or other mammals, and those that are too similar are filtered out. The ones that remain are thus identified as candidate targets for the development of new therapeutic drugs. These genes can be targeted to design and screen effective therapeutic drugs. Compared with traditional drug design strategies, the computational model is more directed, so it can shorten the development time for new drugs (Yu et al., 2020b; Zeng et al., 2020b; Huo et al., 2020; Li et al., 2020; Cheng et al., 2021b; Wang et al., 2021b; Dong et al., 2021). For example, Paul et al. used a biological metabolic network of leishmaniasis and deletion mutations designed using bioinformatics methods to identify a collection of essential proteins for this disease (Ao et al., 2021; Hu et al., 2021b), and this method was more than five times more efficient than the method of randomly selecting potential drug targets (Stanly Paul et al., 2014; Chiu et al., 2020; Wang et al., 2020). As full genome sequences are obtained for more organisms, and with the continuing development of functional genomics research, more attention is being paid to the relationships among genes (Wang et al., 2019), proteins, and phenotypes (Kitano, 2002). Proteins are indispensable components of cellular structures, and participate in a wide range of processes that affect growth, physiology, and development (Eisenberg et al., 2000). In addition, proteins function in particular subcellular compartments (Huh et al., 2003).

### Gene Computation and Prediction Methods Based on Communication Coding Theory

In 1995, Itaya studied the minimum number of chromosomal loci required for the survival of *B. subtilis* using an induced mutation method. Mutations of only six out of 79 randomly selected chromosomal loci prevented the formation of bacterial colonies. Thus, it was inferred that genes at these six loci were essential for the survival of *B. subtilis* (Itaya, 1995). Later, other researchers determined the essential genes of *Mycoplasma* genitalium and explored the composition of the minimum gene set with experimental methods (HutchisonPeterson et al., 1999). Ongoing research on the minimum gene set has continuously discovered new essential genes, thus opening the door to essential gene characterization and further in-depth research.

Hwang et al. studied the topological properties of essential and non-essential genes in protein interaction networks with *Saccharomyces cerevisiae* and *E. coli* as the research objects. Then, they predicted essential genes using machine learning methods and on the basis of protein interaction networks and sequence information (Hwang et al., 2009; Song et al., 2021). Deng et al. used machine learning methods, combined with three types of 8 characteristic parameters such as subcellular localization, phylogenetic information and expression levels, and co-expression networks to predict the essential genes of 4 species including *E. coli*. However, the effectiveness of this method relies on similar feature distributions in the research objects (Deng et al., 2011). Yang et al. studied human essential genes by constructing a classifier based on 28 protein interaction network topological features and 22 biological features (Yang et al., 2014). Yu et al. identified six fractal features of DNA and protein sequences of DEG bacteria, and classified sequences using naïve Bayes and random forest methods. Arun et al. studied the relationships among gene conservation, repetition, constitutive expression, and gene essentiality in *E. coli* (Arun et al., 2016). Although those studies made considerable progress, there were still some problems: few species were studied; the universality of prediction models and analysis results and prediction accuracy needed to be improved; and characteristic parameters that effectively describe essential genes needed to be identified.

Considering that information transmission and coding are similar between biological systems and modern communication systems, communication coding theory can be a useful tool for essential gene analysis and prediction of gene functions. The definition of coding problem in DNA computation was first proposed by Garzon and Deaton et al. in literature network, and then the complete definition of coding problem in DNA computation was given in 2004 after refining and summarizing (Garzon and Deaton, 2004).

The steps involved in essential gene prediction based on the theory of communication coding are as follows: First, a genetic sequence analysis model is constructed based on communication coding theory. The model includes sequence analysis based on simple coding models (such as block codes, convolutional codes) and cascaded and mixed coding models. In error correction coding, convolutional codes (based on channel coding methods but with better performance), have been proven to be as effective as block codes in theory and in practice. The next step is to extract the characteristic parameters that describe essential genes. The combination of the theoretical model of communication coding for analyzing genetic sequences and genetic database information can reveal correlations between information units in DNA sequences at different scales in the coding sense, and extract the characteristic parameters of essential genes. Then, based on the features of the coding meaning and the omics data, the necessary gene calculation and prediction are carried out and combined with the determined gene data in the database, the performance optimization parameters of the established model are evaluated. Finally, the noise or redundancy is removed from the acquired features, and the key features are screened out, which are called “essential features,” and the feature set formed by these “essential features” is called “minimum feature group.” The “minimum feature group” should be constructed within the range of existing features. This will simplify and optimize the model and the analytical process, and improve its analytical efficiency and universality while ensuring prediction accuracy.

### Analysis and Design of Gene Computational Prediction Models Based on Communication Coding Theory

According to the research of gene prediction method based on communication coding theory in 3.1 summary, we use the following research steps to establish a prediction model:

1. Check published studies to understand the current research situation.
2. Collect and preprocess datasets from open databases such as DEG, NCBI, KEGG, and Uniprot, and clean and sort the collected data.
3. Model research and feature extraction. This step includes encoding - based sequence analysis model and feature extraction. 1) Sequence analysis model based on coding; If the genetic sequence is analyzed as a sequence with certain information and coding characteristics, the biological sequence (DNA, RNA, or amino acid sequence) needs to be expressed in a form that is suitable for analysis, regardless of which analytical model is used (Dao et al., 2021). In this context, researchers have proposed digital mapping methods, graphic expression methods, and geometric expression methods. Taking a DNA sequence as an example, the digital mapping method expresses the four bases A, G, C, and T as different values, in a form that is convenient for computer processing, including integer expression and plural expression (Rosen, 2006; Liu et al., 2021a). The graphic and geometric expression methods express each base as a vector in space, and then connect the corresponding vectors to map a curve in the space. These methods allow for visualization of the sequence structure, and the local and overall characteristics of the sequence. Visualization of sequences is also useful for sequence comparisons and sequence similarity analyses. Once the biological sequence has been expressed and visualized, models based on convolutional code and block code models can be designed, adjusted, and modified according to analytical results and biological significance. Models can be analyzed using cascaded codes and mixed codes on the basis of simple model analysis. 2) Extract features of essential genes: According to different observation scales, the basic information units are set as bases, codons, genes, or the whole genome. Then, the association between essential genes and information units is investigated. This results in the extraction of characteristic parameters that reflect the essentiality of genes in combination with the parameters of the coding model (code length, generator matrix, constraint length, and code distance).
4. Computationally predict essential genes: After completing the tasks in Step 3, essential genes are predicted using machine learning algorithms. The best model is selected through performance evaluation. Redundant features are removed and essential features are screened to identify the “minimum feature group.”
5. Conduct performance testing and feedback optimization: Test the analytical performance of the prediction model based on cross validation and other methods (Jiang et al., 2013; Xu et al., 2021), using the essential/non-essential genes listed in DEG and other databases as training samples and verification samples.
6. Develop software to implement analysis and prediction algorithms and visualize the results. Package the final model into an executable program module with a functional interactive webpage. Establish an online service platform to provide online data analysis services.

**FIGURE 1:**
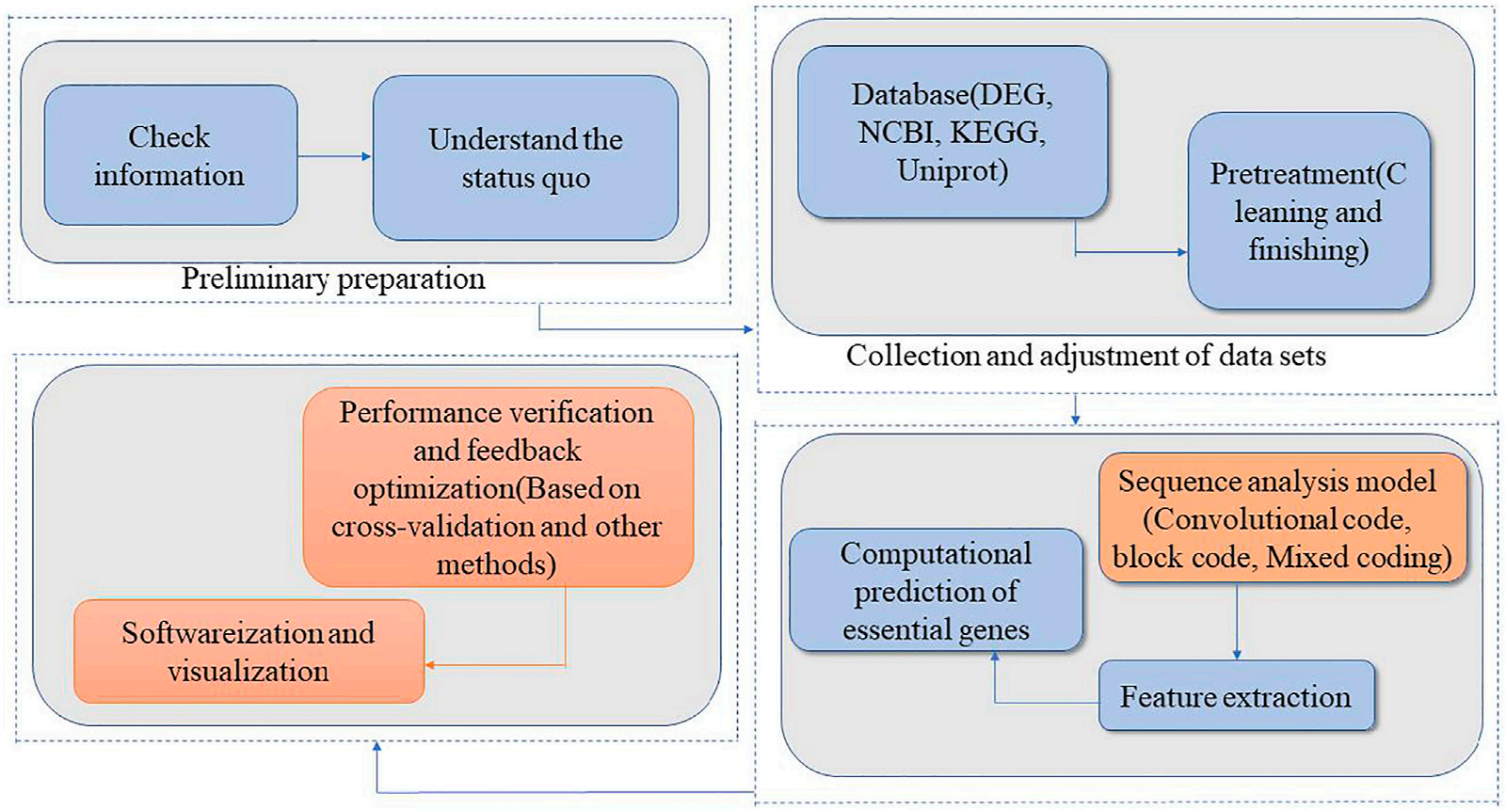
Technical roadmap of gene computational prediction model based on communication coding theory.

The process of the research method is illustrated as a roadmap in Figure 1.

## Key Protein Identification Method

A gene, the basic unit of heredity, is the DNA fragment required to produce a functional RNA or a polypeptide chain that forms into a functional protein. Therefore, genes and proteins are inextricably linked. For example, to explore the characteristics of essential genes, it is necessary to integrate and summarize the genomic metabolome, proteome and other omics data, and extract their combined data features, including metabolic pathways, evolutionary conservation, protein domains, and protein interactions (Wang et al., 2018; Zhang et al., 2018; Chen et al., 2019; Chen et al., 2020b; Yu et al., 2020c). Thus, it is important to identify proteins and collect all relevant information about their sequence, structure, localization, and function.

Protein is the material basis of life, is an organic macromolecule, is the basic organic matter that constitutes cells, and is the main undertaker of life activities (Wei et al., 2014; Wei et al., 2017; Guo et al., 2020; Tao et al., 2020; Wei et al., 2020; Guo et al., 2021). The human body contains many types of proteins with different properties and functions. All proteins have something in common: they are composed of 20 amino acids in different proportions, and they are constantly metabolized and renewed in the body. Many methods for identifying protein complexes based on protein interaction networks have been proposed. Some proteins are essential for the survival and reproduction of organisms, and for essential biological processes. Without them, organisms will have serious defects in their growth and development, or may even die. Thus, these are known as essential proteins. At the same time, research show many proteins perform specific biological functions only after they participate in the formation of protein complexes and interact with other proteins in the complex, suggesting that protein interactions are related to protein complexes. Therefore, this part takes the key proteins as the research object and uses the methods based on the subcellular protein interaction network and subcellular importance to identify the key proteins (Cheng et al., 2021a; Zulfiqar et al., 2021).

The centrality algorithm, a method used to identify key proteins in a protein-protein interaction network, is often used to quantify the contribution of a specific node in the graph and its impact on the network and it’s based on the law of central-lethality (Liu et al., 2020c; Zhai et al., 2020). Nodes with higher centrality values are usually regarded as key nodes in the network. Commonly used graph-based centrality algorithms include degree centrality, betweenness centrality, tight centrality, subgraph centrality, node cluster centrality, and local average connection centrality. However, this method cannot fully assess the criticality of proteins because it does not take temporal and spatial characteristics of protein interactions into account. Therefore, the subcellular location information can be used to construct a subcellular protein interaction network, and a key protein identification method based on the subcellular protein interaction network (LSED) is proposed. LSED is first based on a given global protein interaction network and protein The subcellular location information of each subcellular compartment is constructed to construct a subcellular compartment protein interaction network (PSLIN). Then, its credibility is calculated based on the size of the protein interaction network in each subcellular interval. Next, the centrality scores of the proteins in the subnets of the protein interaction network for each subcellular interval are calculated with a centrality method. Finally, the Localization-specific Centrality Score is calculated for each protein based on its centrality score in the protein interaction network for different subcellular intervals and the credibility of the network. By comparing the prediction accuracy of LSED method on PSLIN with that of centrality method on global protein interaction network, a multi-species average accuracy index (AKAcc) can be proposed, which can more comprehensively evaluate the prediction accuracy of various methods on multiple species. The key protein recognition methods mentioned in this paper are shown in Table 1.

**TABLE 1:** Key protein identification methods.

Methods	Principle
Degree centrality	Based on protein interactions, a reliable PPI network can be constructed by combining other protein biological information. Subsequently, key protein identification is carried out through the centrality method related to network topology
Betweenness centrality	
Tight centrality	
Subgraph centrality	
Node cluster centrality	
Local average connection centrality	
PSLIN	Use subcellular location information to construct a subcellular protein interaction network
LSED	Key protein identification method based on protein interaction network in subcellular compartment
CIC	Centrality method based on the importance of subcellular intervals

Because different subcellular intervals differ in importance, protein interactions in different subcellular intervals also differ in their importance. Therefore, the importance of protein interactions can be estimated based on the importance of subcellular intervals. A Centrality-based Independent Cascade (CIC) based on the importance of subcellular intervals has been proposed to detect essential proteins. For two interacting proteins u and v, the importance of the interaction (u,v) is defined as the maximum importance of the subcellular interval where the two proteins cooccur. A weighted protein interaction network is constructed based on the importance value of the interaction, and the CIC centrality method is used to estimate the criticality of proteins in this network. Thus, the CIC centrality score of a protein depends on the importance of its interactions in different subcellular intervals. Finally, the predictive performance of the CIC method is compared with those of other centrality algorithms. In addition, researchers conducted experiments on the protein interaction network of yeast, human, mouse and fruit fly to compare the prediction performance of CIC method with other centrality methods, including topology-based centrality methods and methods integrating other biological knowledge.

There are close relationships between protein complexes and essential proteins. Studies have shown that the criticality of a protein is not only determined by a single protein node, but often by the function of protein complexes. According to experimental data, essential proteins tend to aggregate in large amounts in certain complexes. Machine learning methods can be used to identify such protein complexes. At present, there is no strict mathematical expression or unified definition for the subgraph model corresponding to protein complexes and functional modules in interaction networks. However, some graph-clustering methods are effective for identifying protein complexes and functional modules, such as the graph-clustering algorithm RNSC, and density-based algorithms such as MCODE. Some studies have found that most proteins in the same protein complex have similar or identical functions, so some hierarchical clustering methods based on similarity or distance are also used to identify protein complexes. Hierarchical clustering algorithm can be divided into two categories, condensation algorithm and splitting algorithm. Among them, the G-N algorithm, a classic splitting algorithm, removes the edge with the highest betweenness and recalculates the betweenness of all edges. When at least two of the generated subgraphs meet the definition of the module after removing the edges, a corresponding phylogenetic tree can be drawn. And the HC-PIN method uses weighted edge clustering coefficient to do fast agglomerative clustering. The clustering idea is to merge the two clusters if there is an edge with the highest edge clustering coefficient between two clusters. At the same time, Table 2 shows various methods for identifying protein complexes.

**TABLE 2:** Identification methods of protein complexes.

Methods	Specific type
Graph clustering method	RNSC algorithm based on graph partition
	Density-based local search algorithm MCODE
Hierarchical clustering method based on similarity or distance	Condensation algorithm
	Division algorithm

## Existing Problems and Research Prospects

In this paper, we have discussed some of the methods used to analyze and process biological sequence information, and introduced the use of coding theory in communication engineering to calculate and predict essential genes. However, some problems are yet to be solved. First, limited data are available for the preliminary analysis of essential genes. The DEG database contains information for a limited number of species, and this makes it difficult to test and validate analytical models. In the identification and function prediction of proteins, it is seldom possible to determine protein function based on a single experiment, because protein function is also affected by environmental factors. In addition, due to various biological, budgetary, or ethical factors, experiments cannot be performed on some organisms. Experiments conducted *in vitro* may not truly reflect the activity of proteins in the body. Moreover, the datasets in biological databases have problems such as noise, deviation, and missing data (Su et al., 2019; Liu et al., 2020d; Liu et al., 2021b; Su et al., 2021).

There are many commonalities between the transmission of genetic information and communication information. Therefore, methods based on communication coding theory can be applied to identify essential genes in bacteria and other organisms. This provides a new perspective and approach for analyzing and predicting essential genes. The information obtained in these analyses has applications in disease prevention, diagnosis, and treatment.
